# Spatial study of dengue and its association with livestock farming in Bantul Regency, Yogyakarta Province, Indonesia

**DOI:** 10.14202/vetworld.2024.2667-2674

**Published:** 2024-11-28

**Authors:** Dila Hening Windyaraini, Raden Wisnu Nurcahyo, Sitti Rahmah Umniyati, Prima Widayani, Suwarno Hadisusanto

**Affiliations:** 1 Department of Tropical Biology, Faculty of Biology, Universitas Gadjah Mada, Yogyakarta, Indonesia; 2 Department of Parasitology, Faculty of Veterinary Medicine, Universitas Gadjah Mada, Yogyakarta, Indonesia; 3 Department of Parasitology, Faculty of Medicine, Public Health, and Nursing, Universitas Gadjah Mada, Yogyakarta, Indonesia; 4 Department of Geographic Information Science, Faculty of Geography, Universitas Gadjah Mada, Yogyakarta, Indonesia

**Keywords:** dengue, domestic animals, livestock, vulnerability

## Abstract

**Background and Aim:**

Dengue fever is a recurring arboviral disease. The presence of livestock and domestic animals potentially increases the risk of dengue fever in an area due to the shared habitats of vectors and humans. Therefore, this study aimed to determine the vulnerability map of dengue disease and identify the influence of livestock and domestic animals on the number of cases in Bantul Regency.

**Materials and Methods:**

An observational study was conducted in 3 *Kapanewon* (subdistricts) in the Bantul regency, known as the dengue-endemic area. The locations of 302 cases were recorded using the Global Positioning System. Dengue case density was analyzed using Kernel Density Estimation, and vulnerability was assessed using an overlay in ArcGIS Desktop 10.8. Furthermore, buffer analysis was conducted to determine the relationship between case density and the presence of livestock and pet pens.

**Results:**

Banguntapan, Kasihan, and Sewon subdistricts had high vulnerability areas of 424.12 Ha (14.97%), 334.76 Ha (10.46%), and 196.12 Ha (7.05%), respectively. The villages with dengue hotspots were Banguntapan and Potorono (Banguntapan Subdistrict) and Tirtonirmolo (Kasihan Subdistrict). The highest number of patients (180 cases) occurred at a buffer distance of <100 m from houses to livestock pens, closely related to the flight distance of *Aedes* spp. mosquitoes, the dengue vector.

**Conclusion:**

The three subdistricts were predominantly characterized by low dengue vulnerability. However, livestock and domestic animal pens are significant risk factors. This information is crucial for effectively controlling and managing dengue disease in Bantul Regency.

## Introduction

Dengue is the fastest-spreading mosquito-borne viral disease in tropical and subtropical areas. In 2019, approximately 5.2 million cases were reported worldwide [1]. Since early 2024, >5 million cases and >200 dengue-related deaths have been recorded [2]. Environmental conditions can affect the breeding of mosquito vectors. Socio-ecological factors can also influence dengue disease. Brady *et al*. [3] have mentioned that environmental factors and community habits greatly influence the incidence of this condition. Variables that determine the incidence of dengue include gender, environment, mobility, non-use of mosquito repellent, house walls, water sources, and settlement density. Meanwhile, the community’s knowledge, attitudes, and habits are considered risk factors [3]. *Aedes aegypti* mosquitoes and *Aedes albopictus*, potential vectors of dengue virus (DENV) [4], are adapted to peri-domestic urban environments where breeding occurs in containers or water reservoirs. Containers around houses, which serve as breeding sites, strongly correlate with increased mosquito density.

A strategy for controlling dengue disease is spatial distribution analysis, which provides information about the spreading pattern across various areas [5]. The distribution models also benefit disease prevention and mitigation strategies, including vector control, large-scale vaccination programs, and healthcare advice for travelers [6]. Mala and Kumar Jat [7] have demonstrated the use of geostatistical techniques to detect and understand the spatial distribution of epidemics. Integrating disease distribution models with reservoir host data is instrumental in paleogeography. Therefore, it is necessary to consider the spatial patterns of reservoir hosts and vectors to determine the distribution of infectious diseases. DENV is found in both humans and mosquitoes. The four DENV serotypes can circulate in dengue-endemic areas (humans serve as reservoir and amplifying hosts and peri-domestic *Aedes* mosquitoes serve as vectors); as well as in the sylvatic cycle (non-human primates [NHP] serve as reservoir hosts and forest-dwelling *Aedes* mosquitoes serve as vectors) [8]. However, primates and domestic animals, which are potential reservoir hosts, serve as habitats for the virus [8]. Several animals, including NHP, bats, pigs, rodents, dogs, bovidae, horses, marsupials, birds, and flies, have been detected for DENV using polymerase chain reaction and serology tests [9]. A study in Malaysia identified antibodies to the virus in wild monkeys [10].Furthermore, RNA of DENV was detected in bats from Hainan Island, China [11]. It is important to acknowledge that the first DENV-2 genome was discovered by Calderón *et al*. [12] in a population of bats from the Caribbean Islands of Colombia. Human activities, such as deforestation for settlements, agriculture, and plantations, can cause wild animals to live closer to humans, potentially increasing the risk of disease transmission.

Domestic animals and livestock play an important role in human life. It is important to acknowledge that domestic animals live and share living spaces with humans and are frequently regarded as family members. Their presence is associated with the transmission of DENV from mosquitoes to humans. Specifically, the presence of animals around homes is strongly related to the blood meal preference of *Aedes* mosquitoes, particularly *Ae. albopictus*. Livestock, a food source for humans, also has the potential to serve as reservoir hosts for dengue disease due to its proximity to the human population. A significant risk factor for dengue transmission is the presence of livestock and domestic animal pens around houses, acting as breeding places for *Aedes* species. At present, study on the relationship between dengue incidence and the presence of livestock and pet pens is still limited both globally [12] and in Indonesia. This study was conducted to determine the relationship of the distance between houses with dengue patients and livestock/domestic animal pens which has never been studied before. This study also aimed to provide a map of the environmental vulnerability of individuals to dengue disease and identify the influence of the presence of livestock and domestic animal pens on the density of cases in Bantul Regency.

## Materials and Methods

### Ethical approval

The study was approved by the Medical and Health Research Ethics Committee of the Faculty of Medicine, Public Health and Nursing, Universitas Gadjah Mada, Yogyakarta, Indonesia (Approval no. KE/FK/1230/EC/2022).

### Study period and location

**Figure-1:**
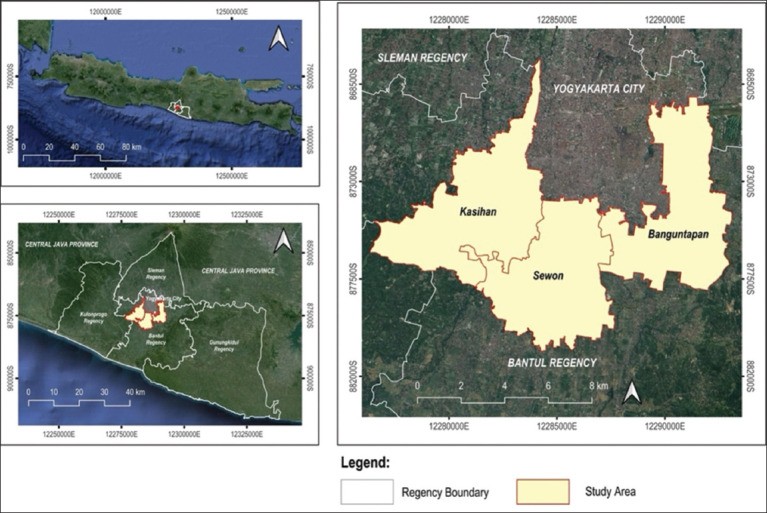
Study area map [Source: The map was generated using ArcMap Desktop 10.8].

The observational study was conducted from January to August 2023, within 3 *kapanewon* (subdistricts) in Bantul Regency: Banguntapan, Sewon, and Kasihan, as shown in Figure-1. These three locations were chosen because they are dengue-endemic areas, with the most cases in Bantul. In addition, these three locations represent urban, rural, and peri-urban areas. The density and mobility of the human population within dengue-endemic areas were also suitable for DENV transmission.

### Data collection

The sampling was conducted in 302 houses across 14 villages within the Banguntapan, Sewon, and Kasihan subdistricts of Bantul Regency. Dengue patient data for 2021–2023 were obtained from the Community Health Center in each area. Furthermore, the geographical coordinates of the patient’s house and the locations of livestock and domestic animal pens were recorded using Global Positioning System (GPS) Garmin GPSMAP 64s (https://www.garmin.co.id/products/discontinued/gpsmap64s-sea/-specsTab).

### Statistical analysis

The obtained GPS data were imported into Microsoft Excel 365 (Microsoft Office, Washington, USA), and the dengue density was analyzed using Kernel Density Estimation with ArcMap Desktop 10.8 (https://www.esri.com/en-us/arcgis/products/arcgis-desktop/overview) In addition, the vulnerability was assessed using ArcGIS Desktop 10.8 (https://www.esri.com/en-us/arcgis/products/arcgis-desktop/overview). The parameters used to map environmental vulnerability were the distance of livestock/domestic animal pens, population density, settlement density, and mosquito flight distance. Buffer analysis was performed to determine the relationship between dengue case density and the presence of livestock and animal pens. Finally, the land cover map was used to determine natural barriers such as rivers, rice fields, and densely populated settlements.

## Results

### Density of dengue

**Figure-2:**
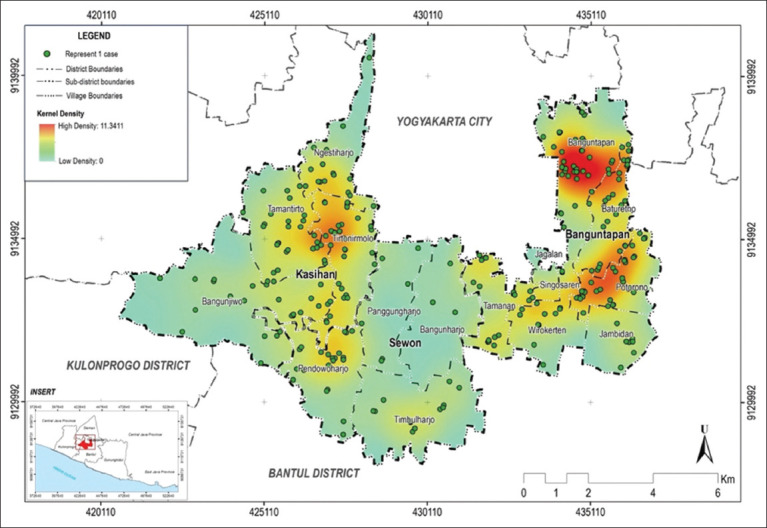
Kernel density analysis of dengue cases in three subdistricts in Bantul during January–August 2023 [Source: The map was generated using ArcMap Desktop 10.8].

Kernel density and buffer analysis showed that only a few villages in three subdistricts had dengue cases. These included Banguntapan and Potorono Village in Banguntapan subdistrict and Tirtonirmolo Village in Kasihan subdistrict, as detailed in Figure-2. The cases were clustered at locations where hotspots were identified. The kernel density analysis showed a density value of 0 to 11.3411 points per square kilometer between 2021 and 2023, with most cases located in settlement areas.

### Area of vulnerability to dengue disease

**Figure-3:**
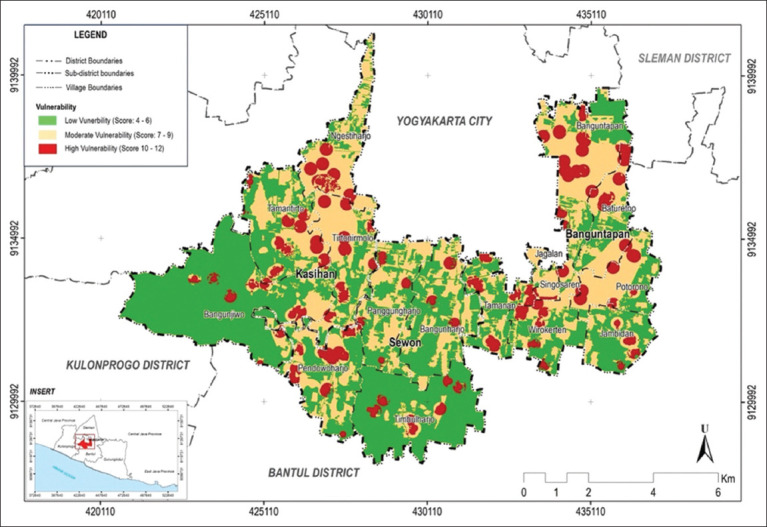
Area vulnerability level to dengue in three subdistricts in Bantul during January–August 2023 [Source: The map was generated using ArcGIS Desktop 10.8].

The three subdistricts had low, medium, and high vulnerability to dengue disease (Figure-3).

**Figure-4:**
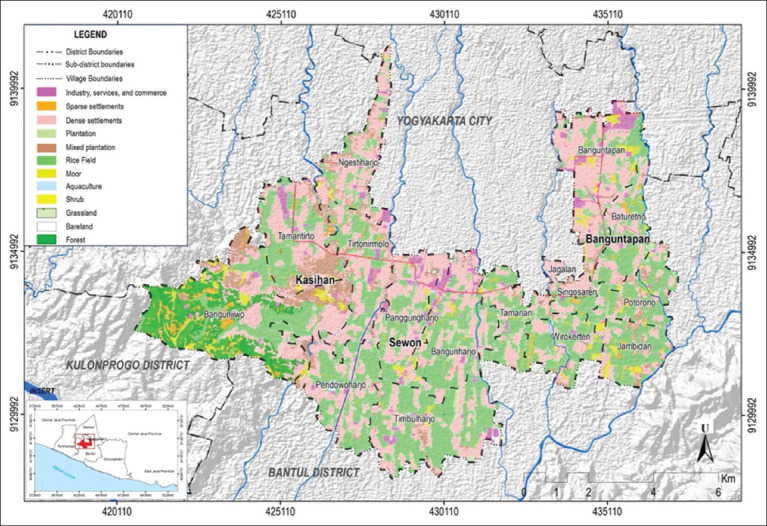
Land cover map in three subdistricts in Bantul [Source: The map was generated using ArcGIS Desktop 10.8].

Areas with high vulnerability (colored red) are distributed throughout the region (Figure-3). A land cover map was adopted to understand land use in the three subdistricts. Areas with high vulnerability were dominated by settlements, rice fields, plantations, bushes, public facilities, industries, and forests. Based on the land cover map, the areas were dominated by densely populated settlements. Low-vulnerability areas are dominated by non-residential land, as shown in Figure-4.

### Relationship between the presence of livestock and domestic animal pens and dengue case density

**Figure-5:**
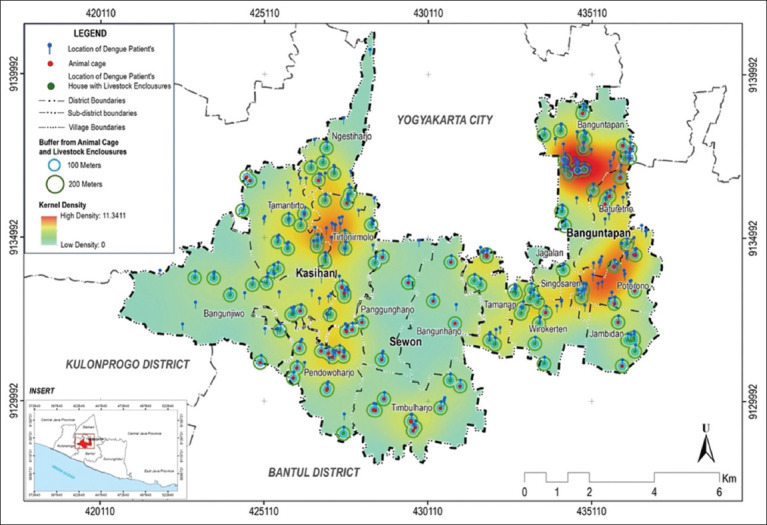
Density and number of dengue cases in livestock and domestic animal pens [Source: The map was generated using ArcGIS Desktop 10.8].

Based on Figure-5, not all patients had livestock or domestic animal pens around their houses. Pens were mostly found in areas with moderate (yellow) to high (orange) dengue case densities. However, they were less frequently detected in areas with lower dengue case densities (green).

**Figure-6:**
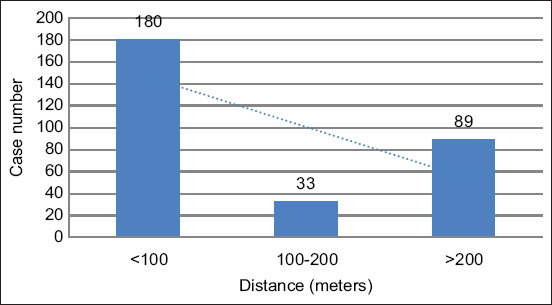
Number of dengue patients based on distance from livestock and domestic animal pens.

The distance between pens and houses was considered to determine the relationship between the presence of livestock and pets with dengue case density. At a distance of <100 m, the number of cases was 180, as shown in Figure-6. For cage distances >200 m, the number of cases was high at 88.

## Discussion

Dengue case density analysis was performed using the Kernel density function in ArcMap Dekstop 10.8 (https://www.esri.com/en-us/arcgis/products/arcgis-desktop/overview). In this process, the density of event locations was calculated based on the estimated kernel density at the pixel center, which was determined by the concentration of events [13]. The surface density on the map represents the density of infections (cases) per km^2^ in the study area. Kernel density analysis is essential for facilitating the government in focusing vector-based disease control programs in dengue case hotspot areas [14].

In the study area, hotspots were detected in three villages: Banguntapan, Potorono, and Tirtonirmolo. Based on the land cover map, these areas were dominated by densely populated settlements, industries, public facilities, and little plantation land. A study conducted in Bangi Town, Malaysia, showed that all dengue cases (100%) were concentrated within infrastructure, utility areas, and housing [14]. The appearance of hotspots can be used as an initial identification result, leading to the hypothesis that the case location proximity related to the sites of growth and development of *Aedes* spp. mosquitoes in the surroundings. Hotspots are regions with high dengue incidence rates. These areas were dengue-prone due to inclusion in the flight range of dengue vector mosquitoes.

The human population density in an area can significantly affect the vector density and dengue incidence [15]. Because humans are hosts of the disease, a high population density can facilitate rapid virus transmission through vector mosquitoes. Urban development, especially in city centers, often leads to the growth of peripheral or satellite areas in terms of population and infrastructure. The three study areas examined are characterized by high population and settlement densities. Many residents in these areas commute to urban centers, such as Yogyakarta and Bantul, resulting in substantial population mobilization. High settlement density can contribute to low sanitation rates, creating slum conditions that are ideal breeding places for mosquitoes. Based on a study by Widayani [16] using geographical information system (GIS) analysis, settlement density and pattern were significant factors associated with dengue incidence.

The dengue incidence is strongly influenced by the vectors in the area, specifically the presence of *Ae. aegypti* and *Ae. albopictus*. The relationship between population and dengue vector densities, especially the number of female *Aedes* spp. mosquitoes, is a crucial risk indicator of dengue transmission in the neighborhood [17] In addition, dengue fever cannot be transmitted directly by human-to-human transmission. Horizontal transmission can be prevented by limiting the presence of mosquitoes as disease vectors [17]. Environmental conditions that support vectorial capacity, either locally or regionally, occur due to enzootic cycles in tropical forests and the experience of inter- and intra-continental spread within the reach of viruses and vectors. Therefore, the presence of many people or dense populations, favorable environmental conditions, and high vector populations lead to the rapid transmission of dengue.

The three subdistricts were dominated by areas with low dengue susceptibility at 52.45% (Table-1). The most vulnerable (high vulnerability) to dengue disease is Banguntapan subdistrict, with an area of 424.12 Ha (14.97%). Low vulnerability indicates that environmental factors are less conducive to virus transmission. Area vulnerability mapping for dengue has also been conducted in other Indonesian regions. It is important to acknowledge that regions with dengue outbreaks, such as Lubuk Linggau City, still have moderate vulnerability (62%) [18].

**Table-1:** Area vulnerability to dengue in three subdistricts in Bantul during January–August 2023.

Vulnerability	Vulnerable area per subdistrict (Ha)						Total size (Ha)

	Banguntapan	%	Kasihan	%	Sewon	%	
Low	1071.65	37.83	1810.43	56.57	1743.03	62.61	4625.11
Moderate	1338.81	47.19	1055.01	32.97	844.62	30.34	3236.44
High	424.12	14.97	334.76	10.46	196.12	7.05	955.00
Total	2832.58	100.00	3200.20	100.00	2783.78	100.00	8.816.55

Meanwhile, Batam Island is still dominated by low vulnerability levels (47.14%) [18] A study conducted in Kendal, Central Java, showed that Kaliwungu and Kaliwungu Selatan subdistricts had low and medium vulnerability despite consistently high dengue incidence rates from 2010 to 2015 [19]. However, the vulnerability of an area to a disease can be influenced by many factors (multifactor), including physical, environmental, and socio-community [20].

Rapid global urbanization has led to the coexistence of humans and animals. This can increase the transmission and spread of vector-borne diseases. According to a previous study by Xuan *et al*. [21], weather and climate are important risk factors for dengue incidence and vector survival in Vietnam. Results showed that farmers had an 8 times higher risk of dengue infection (relative risk: 7.94; 95% confidence interval: 2.29–27.55) [21]. Furthermore, there is a relationship between the presence of DENV-specific immunoglobulin G antibodies and the maintenance of pig health. This may be related to the presence of pig cages, which are a habitat for *Aedes* spp. mosquito larvae [22].

Dengue disease is more commonly observed in urban areas than in rural areas. A study in South Vietnam [22] examined the relationship between Japanese Encephalitis vector mosquitoes and the habits of keeping pigs in urban areas. Results showed that mosquitoes were more prevalent in urban environments with pig farming [23, 24] Furthermore, some studies have shown that the number of mosquitoes is higher in households with livestock.

This study showed that patients’ houses with livestock or domestic animal pens are located in areas with moderate to high dengue case densities. The highest cases (180) were observed in houses situated <100 m from animal pens (Figure-6). For the parameter of pen distance >200 m, the number of cases was 88. This can be attributed to the proximity of residences, prompting the recurrence of the disease within a buffer distance of 200 m.

The presence of livestock and domestic animal pens is closely related to the presence of *Aedes* spp. Animals around houses affect blood meal preference of *Ae. albopictus* mosquitoes. *Ae. aegypti* and *Ae. albopictus* mosquitoes can act as bridge vectors for several diseases. A bridge vector is a key link that connects animals as reservoir hosts to new vertebrate hosts, including humans, in a recurring manner. *Ae. albopictus* mosquitoes have a habit of sucking the blood of mammals, including humans (92%), compared to birds (8%) and other vertebrates (3.7%). Within the scope of mammals, these mosquitoes prefer the blood of humans (60%) and domestic animals, such as dogs, cats, sheep, and cows, to that of wildlife animals [25]

Domestic animals, such as dogs and cats, serve as hosts for some diseases during the rural cycle. This is dangerous because the pathogen multiplies in the body of the amplifying host without the animal showing clinical symptoms of the disease. When a mosquito sucks the blood of a host, it can transmit the pathogen to humans or other animals [25]. This study showed transovarial DENV-3 infection in *Ae. aegypti* and *Ae. albopictus* mosquitoes in three subdistricts. This indicates that DENV-3 is circulating in the human population and other vertebrates in the area. A study in Thailand [26] showed blood serum containing types 2 and 3 DENV in the pet dog population. In an urban area that is a dengue-endemic region, 6/632 (0.95 %) blood samples from poodle breed dogs were positive for DENV. All dogs whose blood samples contained DENV lived in the house with their owners. In addition to domestic animals, imported animals can be infected with the virus. For instance, a study conducted in Egypt [27] identified DENV-specific antibodies in 3/91 camels examined (3.3%). This result is concerning because camels are naturally infected with DENV. Although the virus does not significantly affect the prevalence of dengue disease, imported animals may be at 3 times greater risk of infection compared with local animals [27]

A study by Jakobsen *et al*. [28] in Vietnam reported that animal shelters, gardens, and garbage dumps adjacent to human habitation were associated with increased dengue fever. In this study, there was no relationship between the presence of livestock pens and *Aedes* spp. mosquito larvae. Therefore, further investigation is needed to determine the relationships among livestock management, mosquito-borne infection, and risk factors for dengue disease. Investigating the presence of livestock and domestic animals in dengue-endemic areas is crucial for determining whether these animals are risk factors or act as zoo barriers to DENV in humans.

## Conclusion

GIS was used to create spatial models for an area vulnerability map that identified areas susceptible to dengue. This study revealed that areas with low vulnerability dominated the three subdistricts. However, the presence of livestock and domestic animal pens was a risk factor for dengue incidence. Further investigation is required to determine the role of livestock and domestic animals in disease transmission. This information is crucial for developing effective dengue control and management strategies in Bantul Regency.

## Authors’ Contributions

DHW: Methodology, conceptualization, formal analysis, and drafted and edited the manuscript. SH: Supervised, validated, and edited the manuscript. RWN: Conceptualization, methodology, supervised, validated, and edited the manuscript. SRU: Conceptualization, methodology, supervised, and validated. PW: Methodology and reviewed and edited the manuscript. All authors have read and approved the final manuscript.
